# Validation and psychometric properties of the drug users’ quality of life scale in Iranian population

**DOI:** 10.1186/s13011-020-00289-z

**Published:** 2020-07-22

**Authors:** Sousan Heydarpour, Amir Jalali, Fatemeh Baghaei, Nader Salari

**Affiliations:** 1 Department of Reproductive Health, School of Nursing and Midwifery, Kermanshah University of Medical Sciences, Kermanshah, Iran; 2 Substance Abuse Prevention Research Center, Research Institute for Health, Kermanshah University of Medical Sciences, Kermanshah, Iran; 3 Student Research Committee, School of Nursing and Midwifery, Kermanshah University of Medical Sciences, Kermanshah, Iran; 4 Department of Biostatistics, School of Public Health, Kermanshah University of Medical Sciences, Kermanshah, Iran

**Keywords:** Validity, Reliability, Psychometric, DUQOL, Iranian population

## Abstract

**Introduction:**

Drug dependence and the resultant problems notably decrease the quality of life (QOL). Measuring the QOL in persons who use drugs (PWUD_s_) and planning to improve it can be helpful for rehabilitation programs. Given the absence of a standard tool to measure the quality of life of PWUD, the present study is an attempt to validate psychometric and cultural characteristics of non-injection drug users’ QOL scale.

**Method:**

The study was carried out as a validation and methodological work. The study population consisted of 273 PWUDs in Kermanshah-based drug clinics including outgoing and hospitalized patients. The participants were selected through convenient-quota sampling. After securing the required permission from the copyright owner of the tool, it was forward/backward translated. Face validity and content validity were determined quantitatively and qualitatively. To examine construct validity of the tool, explorative factor analysis and confirmatory factor analysis were used. Internal consistency was measured using Cronbach’s alpha and statistical analyses were performed using SPSS (v.25) and LISREL (v.8).

**Results:**

Explorative factor analysis (EFA) and confirmatory factor analysis (CFA) results supported the tool with one factor and 22 items. The R2 index in the model was equal to 0.99, which means that 99% of the variation of dependent variable (total score of QOL) is attributed to independent variable (22 statements). In other words, 99% of the variation of dependent variable is due to the independent variables in the model. The main indices of the model based on CFA all were higher than 0.9, which indicates goodness of fit of the model (χ2/DF = 2.18, CFI, NFI, TLI = 0.93 GF = 0.84, REMSEA = 0.066, R2 = 0.99). The correlative coefficient was significant (*p* < 0.05). The reliability of the tool based on internal consistency (Cronbach’s alpha) for the subscales ranged from 0.84 to 0.85 and equal to 0.84 for the whole tool.

**Conclusion:**

The Farsi version of non-injection drug users’ QOL scale had acceptable indices and it was applicable to assess QOL in the target population. The tool can be used in different fields of drug addiction.

## Introduction

Chronic and recurrent disorders like drug dependence are a real crisis in one’s life that may attenuate QOL as well [1]. Results have shown that drug addicts have different psychological, physical, social, and emotional needs from healthy individuals [2]. A variety of treatments [3], behavioral changes caused by using drugs [4], problems in relationships with family, friends, and others all affect the QOL in persons who use drugs (PWUDs) [5].

The QOL is one of the key objectives and outcomes in the management and treatment of chronic diseases including drugs abusive use disorders [4]. A higher satisfaction with QOL attenuates the risk of returning to drugs after rehabilitation [3]. The QOL is growingly recognized as an indicator of the outcomes of treatment and health services [6]. The measures of QOL might also help physicians to diagnose specific problems other than the disorders under examination and make better therapeutic decisions [4].

Over the past three decades, measurement of the health-related QOL has become a key parameter to measure clinical outcomes in medical researches [2]. There are different tools to measure QOL in the healthy, disabled, and PWUD. The World Health Organization quality of life (WHOQOL-BREF) is one of the most commonly used tools to measure the different aspects of QOL [7]. Generic instruments evaluate a wide range of the fields of QOL in general population and specific groups of patients [8]. On the other hand, disease/condition-specific instruments are designed to evaluate the problems of a specific group of patients in terms of a specific disorder [9].

Ideally, a QOL tool for PWUDs should not be limited to symptoms and negative reactions to treatments and must encompass the care-seeker’s experiences with daily life as well [10]. In general, QOL in PWUDs is mostly measured using generic instruments [11]. Recently, new tools have been introduced that are specially designed to focus on specific fields of drugs abusive use. Despite the generic instruments, the new tools shed light on the mostly hidden aspects of life in PWUDs [12]. Therefore, using a specially designed tool to measure QOL of PWUD care-seekers can yield accurate information about their conditions in terms of different aspects [13].

Injection Drug Use quality of life scale (IDUQOL) is a tool to measure QOL. It is designed in Canada [13] and used in this study. The original English version contains 17 items [14], which was later increased to 21 items [13]. The psychometric characteristics of the 21-item version was validated in Spain for a sample group with 100 members [15]. Afterwards, a 22-item version was developed to measure general quality of life of drug users in Australia [6]. The 22-item version was named drug users QOL scale (DUQOL) [10]. Given the high number of items and successful use of the tool in different countries, it is expected that the tool can be used in the cultural context of Iran as well. Given the above, the importance of measuring the QOL of PWUD_s_ and the need for a reliable tool in this field, the present paper is an attempt to validate cultural and psychometric characteristics of DUQOL in Iran.

## Methods

### Setting

The study was carried out as a methodological and validating study between Aug. 2018 and June 2019 in Kermanshah – west of Iran.

### Participants

The study was conducted on 300 male non-injection drug users and only 273 questionnaires were used in the study. The mean age of the participants was 34.3 ± 9.06 years and due to incomplete files. The participants were selected through convenient-quota sampling from 22 Kermanshah-based drug clinics. For quota sampling, based on the number of clients treated in each center, and based on the volume of the sample group, the quota percentage of each center was determined and then the number of samples per center was selected based on convenient sampling and the inclusion criteria.

Inclusion criteria were reading and writing literacy, ability to comprehend the questionnaire, no hearing/vision impairment, no severe depression, no physical and mental debilitating disease, and not using synthetic and psychedelic drugs (self-statement and medical file check).

### Instruments

#### Drug Use Quality of Life scale (DUQOL)

The DUQOL specifically measures QOL in PWUD. Hubley and Palepu designed it in 2007 for injection PWUDs [15] and later it was used by other studies for non-injection PWUDs [6]. With 22 statements, the tool covers physical, social, psychological, occupation, and geographical aspects of life. The statements are designed based in Likert’s seven-point scale (1 = very unsatisfied … 7 = highly satisfied). The minimum and maximum scores of the tool are 22 and 154 respectively. There is no statement in the tool with inverse scoring. The total scores of the 22 statements yields the mean score of QOL [15].

#### World Health Organization Quality of Life-BREF (WHOQOL-BREF)

A group of experts designed the WHO quality of life questionnaire (WHOQOL-BREF) in 1996 and the brief version of the tool contains 100 statements. The questionnaire is has four subscales (physical health with seven statements; mental health with six statements; social relationships with three statements; and environment health with eight statements) and a general score. The first two questions are not part of any subscale. The score of each subscale ranges from 4 to 20 so that 4 is interpreted as the worst situation and 20 as the best situation. The scores are converted into standard score from zero to 100 and the higher the score the higher the quality of life [7]. The questionnaire is validated and normalized in Iran with intra-cluster correlation indices of physical, mental, social, and environment health equal to 0.77, 0.77, 0.75, and 0.84 respectively [16].

#### Cultural validation

A common way for normalizing a tool is translation. Here, Wild’s (2005) model was used for translation [17] so that two independent translators first translated the tools into Farsi separately. A panel of study team members examined the translations and a unified version was obtained out of the two translations. Two other translators translated the tool back into English separately. Then the study team members examined the English translations, compared them with the original versions to spot differences, and then ensure comparability of the translations and the original versions. Eventually, the final version of the tool was sent to the designer of the tool for confirmation and feedbacks.

To examine cognitive identicality, the final version was provided to 10 non-injection PWUDs and their ability to comprehend, interpret, and understand the tool was examined. Then, the tool was revised based on the results of cognitive information to ensure cultural compatibility of the tool. Eventual, the revised version was checked for any grammatical and type error and the final version was developed.

To ensure face validity, the tool was provided to 10 care-seekers with reading and writing literacy and through face-to-face interview, they were asked to express their opinions about complicacy of the statements and any ambiguity in the statements.

As to content validity, the tool was provided to 20 researchers, faculty board members, and experts in different fields for examination and modifications. Afterwards, quantitative content validity index was computed for each statement based on Walts and Bassel’s index.

#### Data analysis

To examine construct validity, explorative factor analysis (EFA) and confirmatory factor analysis (CFA) were used [18]. To measure goodness of fit of the model, maximum likelihood method was used and to examine reliability of the tool, internal consistency was obtained using Cronbach’s alpha for each statement and the whole tool. To examine quantitative content validity, content validity ratio (CVR) and content validity index (CVR) were used [19]. Moreover, Kappa coefficient [18] was computed for each statement and concurrent validity of the tool was examined using WHOQOL-BREF.

## Results

Totally, 273 participants were studied. In terms of marital status, 52% of the care-seekers were unmarried; 65.9% did not have a high school; 74.7% lived in urban areas; and 41.4% were freelancers. Moreover, 62.3% lived with their families, 68.9% had less than 100$ monthly income, 66.3% did not have a medical insurance, and 56.8% had consumed drugs for more than 5 years. The rest of demographics is listed in Table 1. In this study CVR, CVI, and Kappa’s coefficient were obtained for each item (Table 2).

**Table 1:** Demographics of the study participants

Variable			N (%)
Marital status		Unmarried	142(50)
		Married	99 (36.3)
		Divorced	32 (11.7)
Educational level		Elementry level	60(22)
		High school	180(65.9)
		Higher Education	33(12.1)
Number of children		None	165(60.4)
		One-Two	89(32.6)
		Three and more	19(7)
Domicile		Urban	204(74.7)
		Suburb	34(12.5)
		Rural area	35(12.8)
Lives		Alone	43(15.8)
		With family	170(62.3)
		With spouse	50(18.3)
		With friends	10(3.7)
Job		Manual worker	98(35.9)
		Freelancer	113(41.4)
		Employed	62(22.7)
Insurance		Yes	92(33.7)
		No	181(66.3)
Income/ monthly		Less than 100$	188(68.9)
		100–300&	68(24.90
		More than 300$	17(6.2)
Drug Use duration		Less than one year	21(7.7)
		1–3 years	40(14.7)
		3–5 years	57(20.9)
		More than five years	155(56.8)
Type of drug	Opiate	Yes	117(42.9)
		No	156(57.1)
	Heroin	Yes	151(55.3)
		No	122(44.7)
	Asian Crack	Yes	20(7.30
		No	253(92.7)
Way of using	Inhalation	Yes	227(83.2)
		No	46(16.8)
	Snuffing	Yes	29(10.6)
		No	244(89.4)
	Oral	Yes	82(30)
		No	191(70)
History of drugs treatment		Yes	212(77.7)
		No	61(22.3)
Numbers of Drugs Treatment		None	62(22.7)
		1–3	102(37.4)
		4–6	58(21.2)
		Seven and more	51(18.7)
Cigarette smoking		Yes	255(93.4)
		No	18(6.6)
Cigarette smoking line /day		None	18(6.6)
		1–10	68(24.9)
		10–20	143(52.4)
		20 and more	44(16.1)

**Table 2:** CVI, CVR, T-value and factor loadings of the tool items

No	Items	CVR^a^	CVI^b^	Kappa Coe.^c^	Mean (SD)	Kurtosis^d^	Skewness^e^	T (cr)^f^	λ^**g**^
1	Being Useful For society and help to people	0.46	0.93	0.93	4.26(1.77)	−0.753	−0.261	9.24	0.55^***^
2	Accessible Community Resources	0.86	0.93	0.93	3.58(1.83)	−0.93	0.145	6.47	0.4^***^
3	Drugs	0.6	0.86	0.86	2.45(1.88)	−0.126	1.035	5.22	0.33^***^
4	Drug Treatment	0.6	0.93	0.93	5.31(1.77)	0.102	−0.98	−4.69	0.30^***^
5	Education and training	0.55	0.86	0.86	3.6(2.01)	−1.205	0.205	7.27	0.44^***^
6	Family	0.75	1	1	4.86(2.12)	−0.945	−0.672	9.07	0.54^***^
7	Feeling Good about Yourself	0.75	0.93	0.93	4.27(1.26)	−1.168	−0.124	10.81	0.62^***^
8	Independence and Free Choice	0.75	1	1	4.7(1.86)	−0.683	−0.598	6.34	0.39^***^
9	Friends	0.75	0.93	0.93	3.44(1.96)	−1.094	0.284	5.28	0.33^***^
10	Caring for Harm Reduction	0.46	0.86	0.86	4.23(1.96)	−1.057	−0.246	6.52	0.4^***^
11	Physical and mental Health	0.46	0.93	0.93	4.3(1.86)	−1.035	−0.14	9.79	0.57^***^
12	Accessible Health Care services	0.46	0.8	0.79	4.6(1.92)	−0.708	−0.557	5.74	0.36^***^
13	Housing	0.46	0.86	0.86	4.4(2.1)	−1.083	−0.473	10.2	0.59^***^
14	How others treat you	0.6	0.93	0.93	3.9(1.79)	−1.03	0.055	11.19	0.64^***^
15	Leisure Activities	0.75	0.86	0.86	3.75(1.75)	−0.77	0.06	8.55	0.51^***^
16	Income/ Money	0.6	0.93	0.93	2.95(1.88)	−0.861	0.587	10.07	0.55^***^
17	Neighborhood Safety	0.46	1	1	4.4(1.97)	−0.998	−0.407	9.56	0.53^***^
18	Partner(s)	0.6	0.93	0.93	2.8(2.7)	−1.22	0.373	6.05	0.32^***^
19	Feeling towards the future	0.6	0.93	0.93	4.46(2.02)	−1.061	−0.104	10.29	0.6^***^
20	Sex	0.6	0.93	0.93	2.07(1.68)	1.191	1.488	5	0.31^***^
21	Spirituality	0.86	1	1	5(1.73)	−0.338	−0.701	6.53	0.4^***^
22	Transportation	0.46	0.93	0.93	3.5(2.14)	−1.318	0.194	8.78	0.52^***^

**a**- Content Validity Ratio

**b**- Content Validity Index

**c**- Modified coefficient Kappa (The coefficient of agreement of experts in CVR & CVI)

**d**- Skewness is a measure of symmetry, or more precisely, the lack of symmetry

**e**-Kurtosis is a measure of whether the data are heavy-tailed or light-tailed relative to a normal distribution

**f**- The calculated values of t for all factor loadings of the first and second order are greater than 1.96 and are therefore significant at the 95% confidence level, g- The specific value, which is denoted by the Lamda coefficient and the statistical symbol λ, is calculated from the sum of the factors of the factor loads related to all the variables of that factor

*** *p* < 0.001

** *p* < 0.01

* *p* < 0.05

Before performing EFA, adequacy of sampling test was conducted to ensure that the sample size is large enough. At first, correlation coefficients of the statements were examined. The KMO test was obtained equal to 0.869 and Bartlett’s test of sphericity was equal to 1471.07. These tests were used to examine suitibility of sample size presumptions for EFA (*p*-value < 0.001). Given that H0 is not supported, a significant relationship between the variables is supported. Therefore, the presumptions of CFA were met and it was conducted on the answers by the subjects to the 22 statements of the scale. Varimax perpendicular rotation and principle component (PC) analysis were used. By examining the overlap of each statements, high overlap of all statements (> 0.5) was supported and thus, none of the statements were removed. A list of extracted components, eigenvalue, and explained variance percentage of each factor are presented in Supplementary Table.

**Fig. 1:**
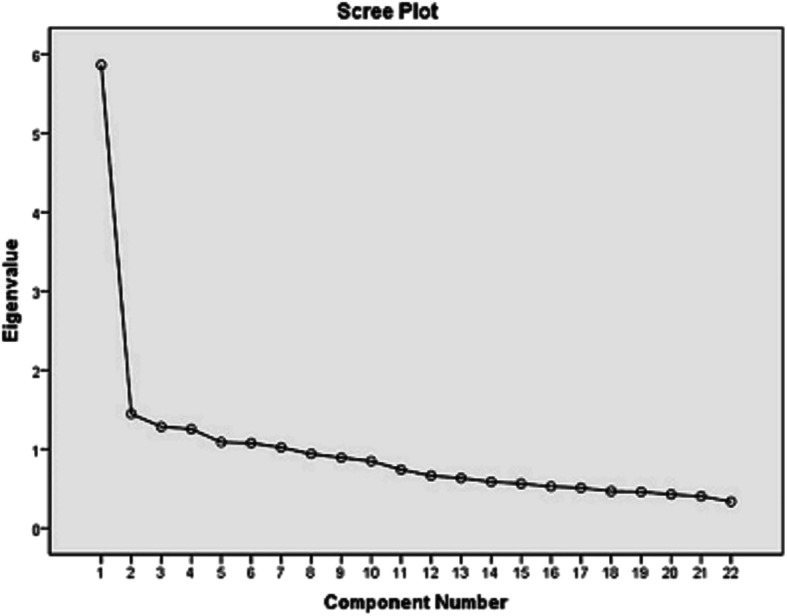
Scree Cattel plot of the extracted elements of the questionnaire

The eigenvalue of second component is 1.44 and ratio of eigenvalues of the first and second components is 4.05. According to [20], when this ratio is higher than 4, the variable has unidimensional factor structure [10]. The screen plot of EFA developed in SPSS (Fig. 1) shows the large difference of the eigenvalue of first component from that of other components.

### Construct validity (CFA)

To examine normal distribution of the variables, KS test was used. With z = 0.645 and *p* = 0.800, the normal distribution of quality of life data of the care-seekers is supported. Moreover, the observed skewness for all statements ranges from 0.004 to 0.648 and within (− 2, 2) interval. This means the distributions of the statements are approximately symmetrical. The value of Kurtosis was from − 1.48 to − 0.302, which is within (− 2, 2) interval.

To examine validity of the model, the *p*-value was less than 0.001 given the factor loadings of each statement. In addition, given the mean value and t-value, the statements were in an acceptable range (Table 2).

**Fig. 2:**
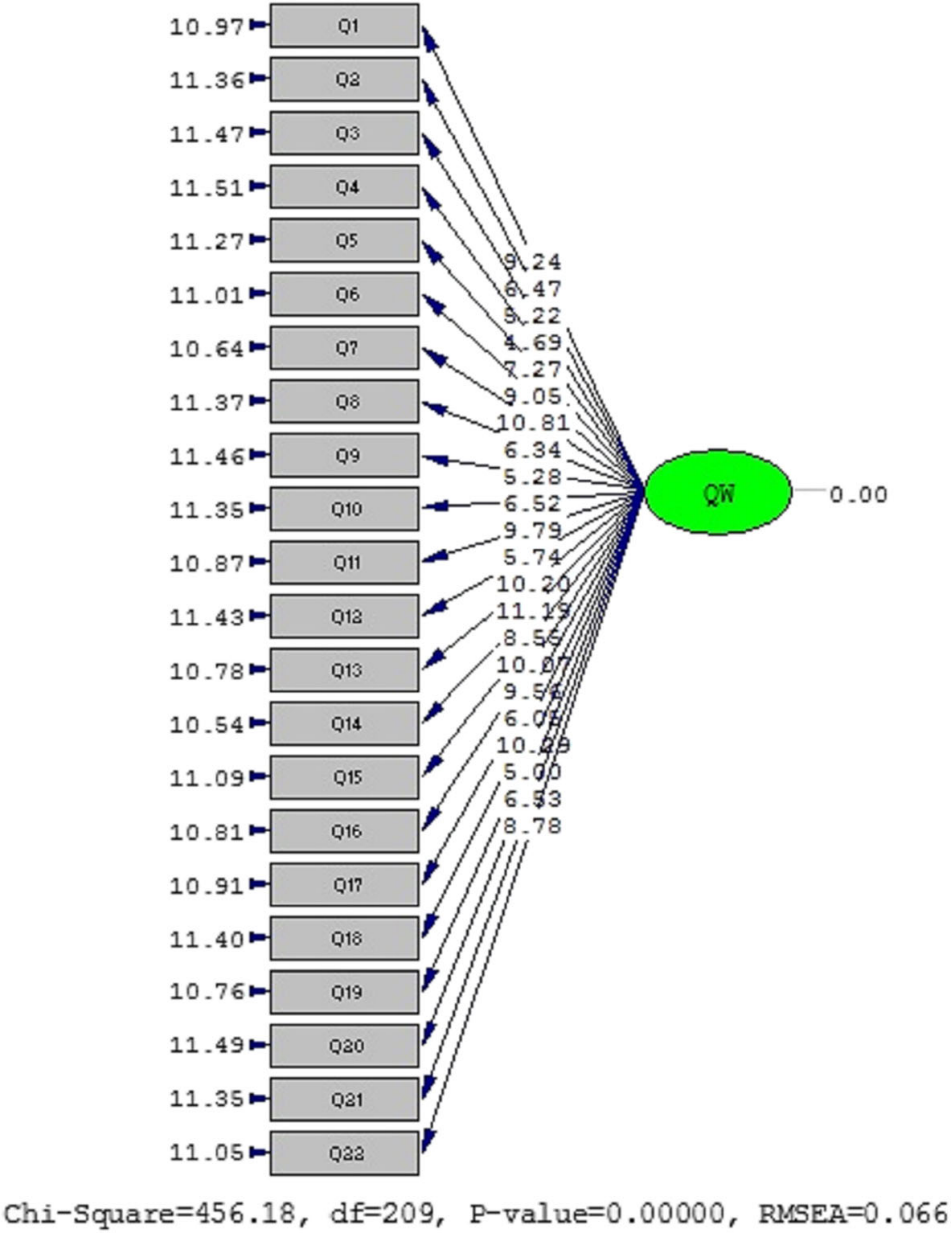
One factor model of DUQOL in Iranian population

Figure 2 illustrates CFA model of the variable under study in two modes of significant and standard coefficients. Given that all t-values are larger than |1.96| and that factor loads > 0.3, none of the statements were removed. Factor analysis results are listed in Table 3. In addition, taking into account the indices of goodness of fit, the goodness of fit of the model with the obtained data is acceptable. All measures of the goodness of fit confirmed that a single factor fit the data well.

**Table 3:** Fit Indicators Confirmatory Factor Analysis Persian Version of DUQOL

Fit Indicators	Criterion	Level	Interpretation
χ^2^/DF	3 ≥	2.18	Optimal fit
CFI	0.9<	0.93	Optimal fit
NNFI/TLI	0.9 <	0.93	Optimal fit
GFI	0.8 <	0.9	Optimal fit
RMSEA	0.08>	0.066	Optimal fit
R^2^	Near to 1	0.99	Optimal fit

To examine internal consistency of the statements with the total score, Pearson’s correlation coefficient was used. The results supported direct and significant correlation of DUQOL statements with the total score of the tool (Table 4).

**Table 4:** Reliability and consistency coefficients of scale of measurement of DUQOL

No	Items	Correlation coefficient		Alpha-Cronbach
		R	*P*-value	
1	Being Useful For society and help to people	0.575	0.001^**^	0.84
2	Accessible Community Resources	0.144	0.017^**^	0.85
3	Drugs	0.356	0.001^**^	0.84
4	Drug Treatment	0.345	0.001^**^	0.85
5	Education and training	0.484	0.001^**^	0.85
6	Family	0.578	0.001^**^	0.84
7	Feeling Good about Yourself	0.65	0.001^**^	0.84
8	Independence and Free Choice	0.433	0.001^**^	0.85
9	Friends	0.377	0.001^**^	0.85
10	Caring for Harm Reduction	0.448	0.001^**^	0.85
11	Physical and mental Health	0.602	0.001^**^	0.84
12	Accessible Health Care services	0.416	0.001^**^	0.85
13	Housing	0.608	0.001^**^	0.84
14	How others treat you	0.617	0.001^**^	0.84
15	Leisure Activities	0.528	0.001^**^	0.84
16	Income/Money	0.507	0.001^**^	0.84
17	Neighborhood Safety	0.619	0.001^**^	0.84
18	Partner(s)	0.591	0.001^**^	0.85
19	Feeling towards the future	0.402	0.001^**^	0.84
20	Sex	0.435	0.001^**^	0.85
21	Spirituality	0.63	0.001^**^	0.85
22	Transportation	0.423	0.001^**^	0.84
	DUQOL			0.85

In addition, Cronbach’s alpha of the whole tool was obtained to check internal reliability of DUQOl (α = 0.84). In the case of subscales, Cronbach’s correlation was in 0.84–0.85 range -i.e. internal reliability of the subscales is supported.

To examine concurrent validity, consistency of DUQOL and WHOQOL-BREF was measured. Pearson’s correlation test supported a direct and significant correlation between DUQOL and WHOQOL-BFEF (r = 0.779, *p*-value = 0.0001). In addition, direct and significant correlations between physical, psychological, and social relationships field with DUQOL were 0.755. 0.442. and 0.546 respectively (*p* < 0.05; Table 5).

**Table 5:** The correlation coefficient of WHOQOL and DUQOL among the subjects

WHO QOL Aspects	QOL Non-injection drug use	
	r	*P*-value
Physical	0.755	0.001^**^
Psychological	0.442	0.001^**^
Social communication	0.546	0.001^**^
QOL (total)	0.779	0.001^**^

** Significant less than 0.01

## Discussion

A Farsi version of DUQOl was validated in Iran. After examining content validity, face validity, reliability, EFA, and CFA, 22 statements remained in the tool.

The EFA results supported construct validity of the 22 statements (χ2 = 1471.072, *P* < 0.0001, KMO = 0.869). Morales-Manrique et al. used EFA for construct validity assessment [10]. The obtained results are consistent with Bartlett’s test of sphericity (KMO = 0.83 ، χ2 = 1099.99, *p* < 000).

Based on the EFA results and maximum varimax rotation, 59.216% of variance was attributed to seven factors. Zubaran et al. [6] used EFA to determine construct and concurrent validity. Factor analysis with maximum varimax rotation probability showed that 59.69% of variance was attributed to six factors.

Pearson correlation test showed a direct and significant relationship between the statements and total score of the scale (the corrected item-total correlations). Zubaran et al. [12] reported that there was a significant correlation between all DUQOL items, which was higher than the internal consistency of the tool. A validating study in Spain [10] also reported a direct and significant correlation in all subscales.

Cronbach’s alpha was obtained equal to 0.71 with variation between 0.7 and 0.8. This indicates reliability of the tool for Iranian population and consistency with Akbari et al. who studied the reliability of quality of life scale in PWUD. Cronbach’s alpha in [21] was obtained equal to 0.87. Morales Manrique et al. reported test-retest correlation of the tool equal to 0.88 [10]. Hubley et al. reported Cronbach’s alpha of IDUQOL scale equal to 0.88, which is consistent with our findings [13].

To examine concurrent validity, consistency of DUQOL with WHOQOL-BREF was examined. Correlation analysis showed a significant correlation between the mean score of DUQOL and the aspects of WHOQOL-BREF. Zubaran et al. also reported a significant correlation between DUQOL scores and the scores of WHOQOL-BREF-BREF with four subscales [6]. Morales-Manrique et al. compared DUQOL and IDUQOL to examine concurrent consistency and reported a direct and significant correlation [10]. The findings are also consistent with Roajs’s et al. results, so that the correlation between the Health-Related Quality of Life for Drug Abusers (HRQOLDA) and WHOQOL-BREF was equal to 0.72 and the correlation between HRQOLDA and DUQOl was equal to 0.61 [22].

Data gathering was through administering the questionnaire and there was no way to examine subjective data of the care-seekers. This a normal limitation of descriptive and validation studies. In addition, female drug users refused to participate in the study despite the explanations given to them. Therefore, the study was limited to male drug users.

## Conclusion

The DUQOL scale is a reliable and valid tool to assess quality of life in drug users in Iran. The tool can be used by applied and health studies.

## Supplementary information

**Additional file 1: Supplementary Table.** Factors extracted after exploratory analysis.

## Data Availability

The datasets used and analyzed during the current study are available from the corresponding author on reasonable request.

## Abbreviations

QOL: Quality of Life
PWUD: Person/s with use/s drug/s
WHOQOL: World Health Organization Quality of Life
CVI: Content Validity Index
CVR: Content Validity Ratio
KMO: Kaiser Meyer Olkin
EFA: Explorative factor analysis
TLI: Tucker-Lewis Index
NFI: Normed Fit Index
GFI: Goodness of Fit Index
CFA: Confirmatory Factor Analysis
RMSEA: Root Mean Square Error of Approximation
PC: Principle Components
HRQOLDA: Health-Related Quality of Life for Drug Abusers
KUMS: Kermanshah University of Medical Sciences

## References

[1] Vederhus J-K, Pripp AH, Clausen T (2016). Quality of life in patients with substance use disorders admitted to detoxification compared with those admitted to hospitals for medical disorders: follow-up results. Subst Abus.

[2] Karow A, Reimer J, Schäfer I, Krausz M, Haasen C, Verthein U (2010). Quality of life under maintenance treatment with heroin versus methadone in patients with opioid dependence. Drug Alcohol Depend.

[3] Pasareanu AR, Opsal A, Vederhus J-K, Kristensen Ø, Clausen T (2015). Quality of life improved following in-patient substance use disorder treatment. Health Qual Life Outcomes.

[4] Muller AE, Skurtveit S, Clausen T (2016). Many correlates of poor quality of life among substance users entering treatment are not addiction-specific. Health Qual Life Outcomes.

[5] Assari S, Jafari M, Preedy VR, Watson RR (2010). Quality of life and drug abuse. Handbook of disease burdens and quality of life measures.

[6] Zubaran C, Emerson J, Sud R, Zolfaghari E, Foresti K (2012). The application of the drug user quality of life scale (DUQOL) in Australia. Health Qual Life Outcomes.

[7] Yousefy AR, Ghassemi GR, Sarrafzadegan N, Mallik S, Baghaei AM, Rabiei K (2010). Psychometric properties of the WHOQOL-BREF in an Iranian adult sample. Community Ment Health J.

[8] Coons SJ, Rao S, Keininger DL, Hays RD (2000). A comparative review of generic quality-of-life instruments. Pharmacoeconomics.

[9] Ruggeri M, Gater R, Bisoffi G, Barbui C, Tansella M (2002). Determinants of subjective quality of life in patients attending community-based mental health services. The South-Verona outcome project 5. Acta Psychiatr Scand.

[10] Morales-Manrique CC, Valderrama-Zurián JC, Castellano-Gómez M, Aleixandre-Benavent R, Palepu A (2007). Cross cultural adaptation of the Injection Drug User Quality Of Life Scale (IDUQOL) in Spanish drug dependent population, with or without injectable consumption: Drug User Quality of Life Scale-Spanish (DUQOL-Spanish). Addict Behav.

[11] Castillo I, Poo M, Alonso I (2004). Evaluation of the SF-36 health index applied to methadone maintenance program users. Reference values for the Basque autonomous community, Spain. Valores de referencia Para la Comunidad Autonoma Vasco. Rev Esp Salud Publica.

[12] Zubaran C, Foresti K (2009). Quality of life and substance use: concepts and recent tendencies. Curr Opin Psychiatry.

[13] Hubley AM, Russell LB, Palepu A. Injection Drug Use Quality of Life scale (IDUQOL): a validation study. Health Qual Life Outcomes. 2005;3.43(1). 10.1186/1477-7525-3-43. 10.1186/1477-7525-3-43 PMC1200562 16029504

[14] Brogly S, Mercier C, Bruneau J, Palepu A, Franco E (2003). Towards more effective public health programming for injection drug users: development and evaluation of the injection drug user quality of life scale. Subst Use Misuse.

[15] Hubley AM, Palepu A (2007). Injection Drug User Quality of Life Scale (IDUQOL): Findings from a content validation study. Health Qual Life Outcomes.

[16] Nedjat S, Montazeri A, Holakouie K, Mohammad K, Majdzadeh R (2008). Psychometric properties of the Iranian interview-administered version of the World Health Organization's quality of life questionnaire (WHOQOL-BREF): a population-based study. BMC Health Serv Res.

[17] Wild D, Grove A, Martin M, Eremenco S, McElroy S, Verjee-Lorenz A (2005). Principles of good practice for the translation and cultural adaptation process for patient-reported outcomes (PRO) measures: report of the ISPOR task force for translation and cultural adaptation. Value Health.

[18] Soeken KL, Waltz CF, Strickland OL, Lenz ER (2010). Measurement in nursing and Health Research. Measurement in nursing and Health Research.

[19] Halek M, Holle D, Bartholomeyczik S (2017). Development and evaluation of the content validity, practicability and feasibility of the innovative dementia-oriented assessment system for challenging behaviour in residents with dementia. BMC Health Serv Res.

[20] Hattie J. An empirical study of various indices for determining unidimensionality. Multivariate Behavior Research. 1984;19(1):49–78. 10.1207/s15327906mbr1901_3. 10.1207/s15327906mbr1901_3 26776067

[21] Akbari B, Andalib M, Andalib S, Khakbiz K, Safarpour H (2012). Reliability and factor analysis of WHOQoL-100 questionnaire for drug addicts in Guilan, Iran. J Basic Appl Sci Res.

[22] Rojas AJ, Lozano O, Foresti K, Zolfaghari E, Zubaran C (2015). Comparison and concordance of health-related quality of life tests among substance users health and quality of life outcomes.

