# Supplementary material for: Validation and psychometric properties of the drug users’ quality of life scale in Iranian population
Source: Subst Abuse Treat Prev Policy. 2020 Jul 22;15:48. doi: 10.1186/s13011-020-00289-z (PMC7374960; doi:10.1186/s13011-020-00289-z)
Supplement: Supplementary file 1 — Additional file 1: Supplementary Table. Factors extracted after exploratory analysis. [file 13011_2020_289_MOESM1_ESM.docx]

Supplementary Table. Factors extracted after exploratory analysis

| Component | Initial Eigenvalues | | | Extraction Sums of Squared Loadings | | |
| --- | --- | --- | --- | --- | --- | --- |
|  | Total | % of Variance | Cumulative % | Total | % of Variance | Cumulative % |
| 1 | 5.865 | 26.657 | 26.657 | 5.865 | 26.657 | 26.657 |
| 2 | 1.445 | 6.569 | 33.226 | 1.445 | 6.569 | 33.226 |
| 3 | 1.283 | 5.832 | 39.058 | 1.283 | 5.832 | 39.058 |
| 4 | 1.253 | 5.694 | 44.752 | 1.253 | 5.694 | 44.752 |
| 5 | 1.087 | 4.939 | 49.691 | 1.087 | 4.939 | 49.691 |
| 6 | 1.076 | 4.889 | 54.580 | 1.076 | 4.889 | 54.580 |
| 7 | 1.020 | 4.636 | 59.216 | 1.020 | 4.636 | 59.216 |

Lists extracted components, eigenvalue, and explained variance percentage of each factor. Clearly, eigenvalue of the first component is 5.86 and 26.65% of variance in the participants’ responses in attributed to this component.
